# Metabolomics Reveals Process of Allergic Rhinitis Patients with Single- and Double-Species Mite Subcutaneous Immunotherapy

**DOI:** 10.3390/metabo11090613

**Published:** 2021-09-09

**Authors:** Peiyan Zheng, Guanyu Yan, Yida Zhang, Huimin Huang, Wenting Luo, Mingshan Xue, Na Li, Jian-Lin Wu, Baoqing Sun

**Affiliations:** 1 Department of Allergy and Clinical Immunology, National Center for Respiratory Medicine, The First Affiliated Hospital of Guangzhou Medical University, National Clinical Research Center for Respiratory Disease, State Key Laboratory of Respiratory Disease, Guangzhou Institue of Respiratory Health, Guangzhou 510120, China; gdmcslxx@126.com (P.Z.); sharpgyyan@126.com (G.Y.); xiaoda0610@163.com (Y.Z.); huanghuimin311@126.com (H.H.); xveyin@163.com (W.L.); acklandfrog@163.com (M.X.); 2 State Key Laboratory for Quality Research in Chinese Medicines, Macau University of Science and Technology, Macao 999078, China; nli@must.edu.mo

**Keywords:** allergic rhinitis, allergen immunotherapy, hydroxyeicosatetraenoic acid

## Abstract

Allergen immunotherapy (AIT) is the only treatment that can change the course of allergic diseases. However, there has not been any research on metabolic reactions in relation to AIT with single or mixed allergens. In this study, patients with allergic rhinitis caused by *Dermatophagoides pteronyssinus* (*Der p*) and *Dermatophagoides farinae* (*Der f*) were treated with single-mite (*Der p*) and double-mite (*Der p*:*Der f* = 1:1) subcutaneous immunotherapy (SCIT), respectively. To compare the efficacy and the dynamic changes of inflammation-related single- and double-species mite subcutaneous immunotherapy (SM-SCIT and DM-SCIT), we performed visual analogue scale (VAS) score, rhinoconjunctivitis quality of life questionnaire (RQLQ) score and serum metabolomics in allergic rhinitis patients during SCIT. VAS and RQLQ score showed no significant difference in efficacy between the two treatments. A total of 57 metabolites were identified, among which downstream metabolites (5(S)-HETE (Hydroxyeicosatetraenoic acid), 8(S)-HETE, 11(S)-HETE, 15(S)-HETE and 11-hydro TXB2) in the ω-6-related arachidonic acid and linoleic acid pathway showed significant differences after approximately one year of treatment in SM-SCIT or DM-SCIT, and the changes of the above serum metabolic components were correlated with the magnitude of RQLQ improvement, respectively. Notably, 11(S)-HETE decreased more with SM-SCIT, and thus it could be used as a potential biomarker to distinguish the two treatment schemes. Both SM-SCIT and DM-SCIT have therapeutic effects on patients with allergic rhinitis, but there is no significant difference in efficacy between them. The reduction of inflammation-related metabolites proved the therapeutic effect, and potential biomarkers (arachidonic acid and its downstream metabolites) may distinguish the options of SCIT.

## 1. Introduction

Allergic rhinitis (AR), also known as anaphylactic rhinitis, mainly refers to non-infectious inflammatory diseases of the nasal mucosa caused by exposure to atopic allergens [1]. A large-scale clinical epidemiological survey of allergens across China found that house dust mites (HDM) are the most prevalent allergens in patients with rhinitis and/or asthma in the south of China, and the sensitization rate increased from north to south, indicating that HDM is an important risk factor for allergic diseases [2,3]. The majority of patients with HDM are co-sensitized or cross-reactive between *Dermatophagoides pteronyssinus* (*Der p*) and *Dermatophagoides farina* (*Der f*) [4]. The international consensus on allergy immunotherapy declared that allergen immunotherapy (AIT) is the only treatment that can change the course of allergic diseases and the only potential disease-modifying treatment for allergic subjects [5]. Studies have shown that both subcutaneous immunotherapy (SCIT) and sublingual immunotherapy (SLIT) could improve allergic disease symptoms and reduce the need for medications [6,7,8,9,10,11], improve the quality of life of patients [12,13], reduce the risk of asthma in patients with AR alone and prevent from being sensitized to further allergens [14].

Regarding the selection of cross-reactive allergens as immune agents in AIT, the World Health Organization and the European Academy of Allergy and Clinical Immunology have declared that selecting a vaccine with mixed or single allergens for immunotherapy would not produce an actual difference, because the two allergens have the same antigens epitope and bind to the same specific IgE antibody, which causes the same allergic reaction [15], and major sensitization proteins with over 80% similarity in the sequence [16]. Conversely, the American Academy of Allergy, Asthma and Immunology (AAAAI) recommends that it may be imperative to limit the allergen types used as immune agents during immunotherapy, with cross-allergens causing the same immune response, based on the consideration of adverse reactions, allergen dilution effects and curative effects, so that a single antigen can be more effectively achieved. Therefore, selecting one type with a cross-reaction antigen may be sufficient [17]. However, no comparative study is currently available on the clinical efficacy and inflammatory response of HDM single-allergen immunotherapy and HDM mixed-allergen immunotherapy.

Metabolomics is an emerging discipline that combines advanced high-throughput analysis techniques, such as mass spectrometry and nuclear magnetic resonance (NMR) spectroscopy, and bioinformatics to study the changes of metabolites within organisms, and it is characterized by high throughput, sensitivity and specificity. Previous metabolomic studies on allergic diseases mainly indicated that the inflammatory metabolic pathway of arachidonic acid (AA) is closely associated with allergic asthma [18,19,20]. AA is metabolized by cyclooxygenase (COX), lipoxygenase (LOX) and cytochrome P450 (CYP450) enzymes. In the pathway, the imbalance of cysteinyl leukotrienes (CysLTs) and lipoxins (LXs), and the proportion of LOX enzyme metabolites, leads to the failure of normal bronchoscopic relaxation, which has been identified as the major factor in the pathogenesis of asthma [21]. Therefore, the leukotrienes regulator was recommended by GINA as a medicine for long-term control of asthma.

5-Hydroxyeicosatetraenoic acid (5-HETE) is a product of the metabolic AA pathway of LOX enzymes. Although it possesses only weak biological activity itself, 5-HETE can be oxidized to 5-oxo-6,8,11,14-eicosatetraenoic acid (5-oxo-ETE), which is a potent chemoattractant for eosinophils and neutrophils [22] and a major metabolite of enzymatic oxidation of AA. 5-oxo-ETE may be an important mediator in asthma, and an attractive target in eosinophilic diseases, such as AR and asthma [23,24]. Several lines of evidence indicate that respiratory infection and cell injury may activate the 12/15-LOX pathway in airway epithelial cells, and markedly increase their production of AA, 12-S-HETE and 15-HETE, which play a pathophysiological role in asthma [25,26]. 

This is a prospective study on AR patients simultaneously sensitized by *Der p* and *Der f* with single-mite subcutaneous immunotherapy (SM-SCIT) or double-mite subcutaneous immunotherapy (DM-SCIT), through the visual analogue scale (VAS) score and rhinoconjunctivitis quality of life questionnaire (RQLQ) score to evaluate and compare the clinical efficacy. On the other hand, we used a specific and sensitive derivatization method combined with ultra-high-performance liquid chromatography-quadrupole-time-of-flight mass spectrometry (UHPLC-Q-TOF/MS) to analyze the differences between serum metabolites and to investigate the changes in eicosanoid metabolism in AR patients during SM-SCIT and DM-SCIT.

## 2. Results

### 2.1. Patients

**Figure 1:**
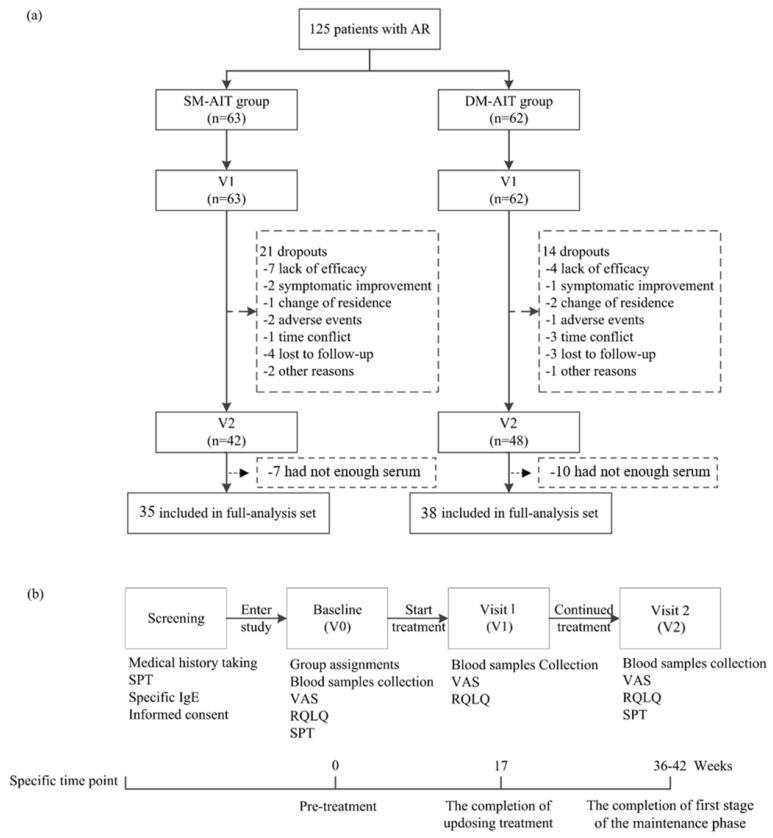
Trail profile (**a**) and flow chart (**b**).

A total of 125 AR patients received SCIT (63 patients were allocated to the SM-SCIT group, while 62 patients were allocated to the DM-SCIT group). Twenty-one patients in the SM-SCIT group and fourteen patients in the DM-SCIT group withdrew during the follow-up, corresponding to dropout rates of 34.3% and 22.2%, respectively. Furthermore, seven patients in the SM-SCIT group and ten in the DM-SCIT group who did not have enough serum (less than 50 μL) for metabolomics approaches in one of the following periods were excluded. Finally, 73 patients (35 in the SM-SCIT group and 38 in the DM-SCIT group) were included (Figure 1). No noticeable differences were found for age, sex, family history of allergic diseases, symptom score, classification of severity, SPT or sIgE between the two groups at baseline (Table 1), and no significant differences in the characteristics between withdrawal groups and protocol groups were found at baseline (Supplementary Table S1).

### 2.2. Clinical Efficacy

**Figure 2:**
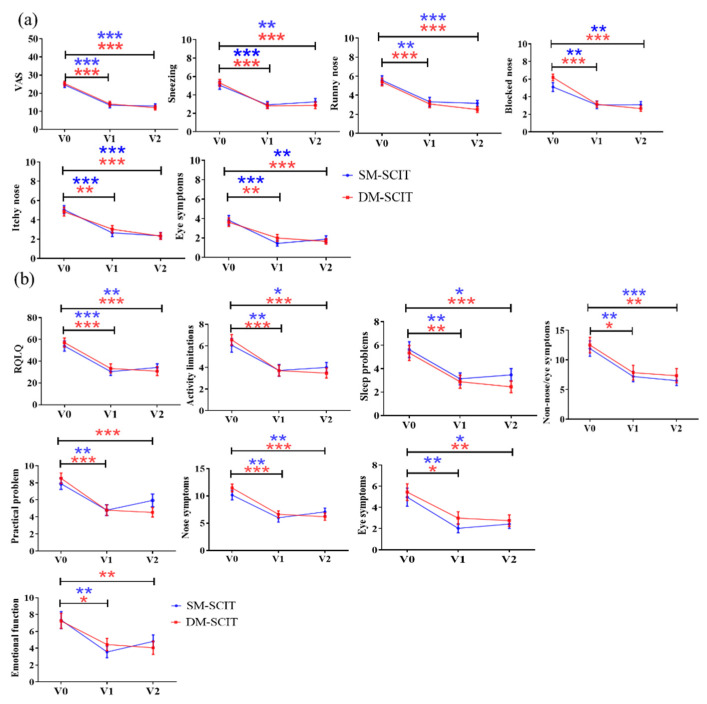
Comparison of two groups of questionnaire scores. (**a**) VAS scores. (**b**) RQLQ scores. Blue, SM-SCIT group; red, DM-SCIT group. All results were expressed as mean ± SEM (standard error of measurement). *, *p* < 0.05; **, *p* < 0.01; ***, *p* < 0.001.

The overall VAS scores and specific clinical symptoms, such as sneezing, blocked nose, runny nose, itchy nose and eye symptoms, were significantly decreased from baseline (V0) to the completion of initial treatment (V1) and the first stage of maintenance treatment (V2) in both SM-SCIT and DM-SCIT groups (*p* < 0.01). However, overall VAS scores, runny nose and itchy nose were significantly decreased between V1 and V2 in the DM-SCIT group. Moreover, no significant differences were found in the overall VAS scores or the five specific symptoms between the two groups during follow-up (Figure 2a). The overall total RQLQ scores and activity limitations, sleep problems, non-nose/eye symptoms, practical issues, nose symptoms, eye symptoms and emotional function at V1 and V2 were significantly decreased compared to V0 in both groups (*p* < 0.01). There were no significant differences in RQLQ scores and the seven domain scores in V0, V1 and V2 between the two groups (Figure 2b).

### 2.3. Metabolomics Analysis of Potential Systemic Biomarkers in AR Patients with SM-SCIT or DM-SCIT

To understand the dynamic changes of anti-inflammatory and pro-inflammatory metabolites in AR patients during SCIT, we performed a metabolomics analysis and multivariate analysis of the serum in patients with SM-SCIT and DM-SCIT.

**Figure 3:**
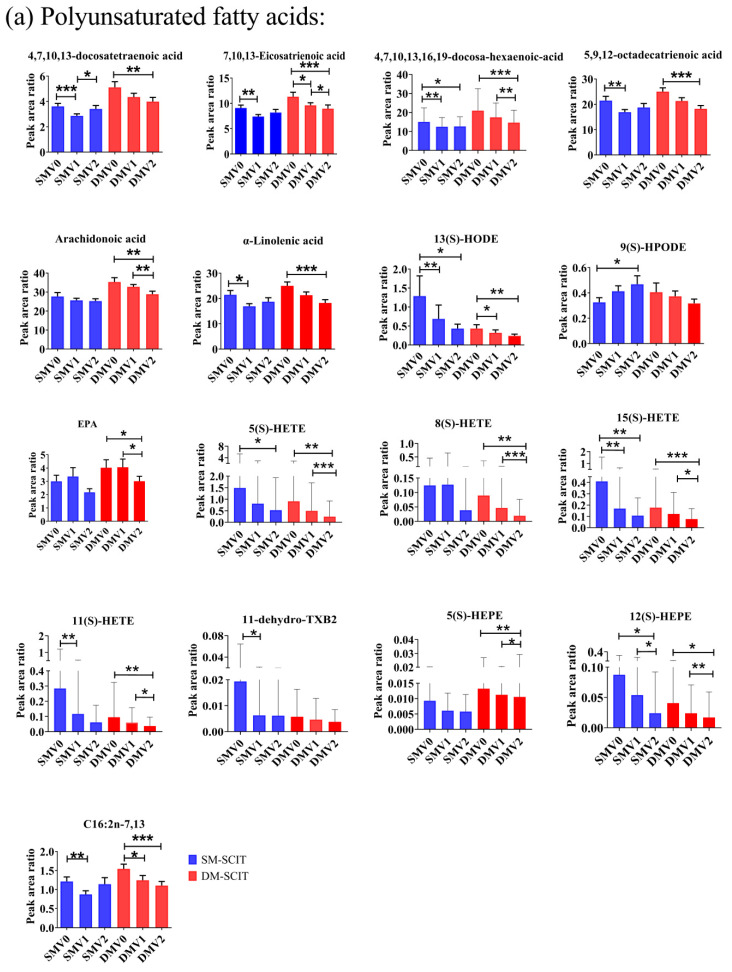

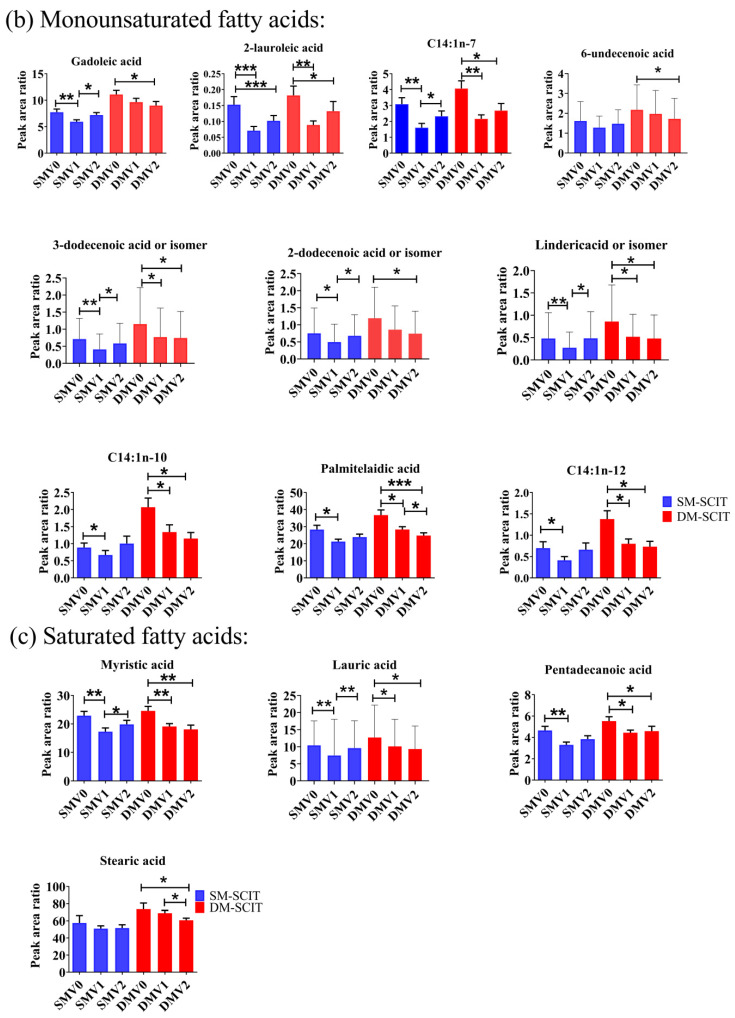
Comparison of the concentration of metabolites in patients: (**a**) polyunsaturated fatty acids metabolites, (**b**) monounsaturated fatty acids metabolites and (**c**) saturated fatty acids metabolites. Blue column, SM-SCIT; red column, DM-SCIT. All results were expressed as mean ± SD (standard deviation), *, *p* < 0.05; **, *p* < 0.01; ***, *p* < 0.001.

**Figure 4:**
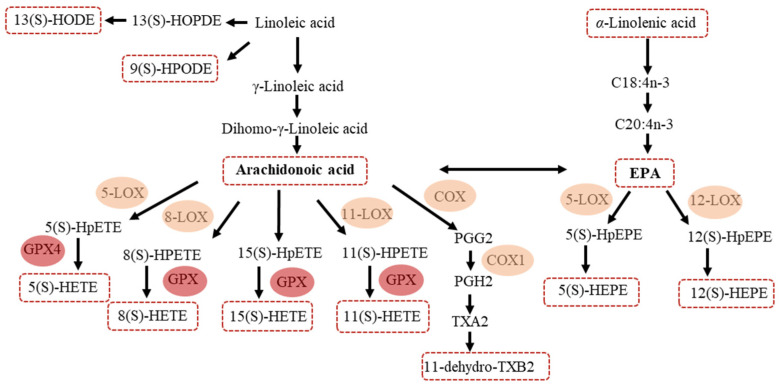
Eicosanoid metabolism pathway in patients with DM-SCIT and SM-SCIT.

The targeted metabolomic approach was used, which was reported in our previous research [27], and a total of 57 metabolites were identified and relatively quantified in serum of AR patients with SM-SCIT or DM-SCIT. Samples within V0 groups were separated from V2 groups using orthogonal partial least squares discrimination analysis (OPLS-DA) (DM-SCIT: R2 = 0.659, Q2 = 0.026; SM-SCIT: R2 = 0.427, Q2 = −0.0352) models (Supplementary Figure S2). Furthermore, 31 metabolites were found significantly decreased by t-test from V0 to V1 and V2, and 12 metabolites were shown according to their metabolism pathways (Figure 3 and Figure 4).

As a result, 15(S)-HETE, 5(S)-HETE, 12(S)-HEPE and 13-HODE were significantly lower after treatment, both in SM-SCIT and DM-SCIT patients. 11(S)-HETE, 8(S)-HETE, 5(S)-HEPE, AA and EPA decreased in DM-SCIT patients, and only 9(S)-HPODE increased in SM-SCIT patients (Table 2 and Figure 3a). Further analysis revealed that there were 5 downstream metabolites (5(S)-HETE, 8(S)-HETE, 11(S)-HETE, 15(S)-HETE and 11-hydro TXB2) in the ω-6-related AA and linoleic acid pathway that showed significant differences, but there was no significant change of 8(S)-HETE after SM-SCIT and in 11-hydro TXB2 after DM-SCIT. Moreover, the ω-3-related α-linolenic acid pathway including its downstream metabolites 5-HEPE and 12-HEPE exhibited significant differences after DM-SCIT, and 12-HEPE was significant decreased after SM-SCIT (Figure 3a and Figure 4).

In addition, Figure 3 showed that 19 kinds of metabolites in another pathway changed during SCIT, including polyunsaturated fatty acids metabolites (5 metabolites: 5,9,12-octadecatrienoic acid, 4,7,10,13,16,19-docosahexaenoic acid, 4,7,10,13-docosatetraenoic acid, 7,10,13-eicosatrienoic acid and C16:2n-7,13), monounsaturated fatty acids metabolites (10 metabolites: 2-lauroleic acid, 3-dodecenoicacid, 2-dodecenoicacid, linderic acid, C14:1N-7, C14:1N-10, C14:1N-12, gadoleic acid, 6-undecenoic acid and palmitelaidic acid) and saturated fatty acids metabolites (4 metabolites: myristic acid, pentadecanoic acid, stearic acid and lauric acid).

### 2.4. The Change Degree of Metabolites during SM-SCIT and DM-SCIT

**Figure 5:**
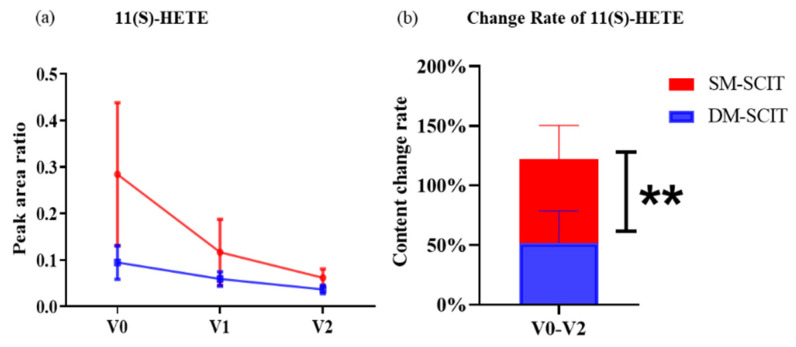
Analysis of the change degree of metabolic components. (**a**) Comparison of the concentrations of 11(S)-HETE between DM-SCIT and SM-SCIT groups from the pre-treatment stage (V0) to the first stage of the maintenance phase (V2). The results were expressed as mean ± SEM. (**b**) Comparison of the change rate of 11(S)-HETE concentrations between DM-SCIT and SM-SCIT groups at V0 and V2. Blue, DM-SCIT group; red, SM-SCIT group. **, *p* < 0.01. The results were expressed as mean ± SD (standard deviation).

In order to distinguish the anti-inflammatory and proinflammatory levels between SM-SCIT and DM-SCIT, we used the ratio of changes in metabolites’ levels to study the degree of metabolite changes during treatment. In particular, the degree of change of 11(S)-HETE in AR patients with SM-SCIT was significantly different from DM-SCIT (Figure 5), indicating that the content of this component decreased more in patients with SM-SCIT.

### 2.5. Association between Biomarkers and Clinical Response

A correlation heat map was performed to evaluate the relationship between the changes (Δ: post-treatment minus pre-treatment) in metabolic components and the improvement in VAS and RQLQ score (∆VAS and ∆RQLQ) in Supplementary Figure S1. No significant correlations between baseline patient characteristics and efficacy of SM-SCIT and DM-SCIT were observed. Variables with a *p*-value of <0.01 (from Supplementary Table S3) in the ∆overall RQLQ and the potential candidate biomarkers in metabolic components are included in Table 3. Correlation analysis revealed that serum metabolic components (including 8(S)-HETE, 13-HODE or isomer, 11-dehydro TXB2, 5(S)-HETE, 15(S)-HETE and 11(S)-HETE) were associated with an improvement in RQLQ (*r* = 0.424, 0.418, 0.374, 0.363, 0.360 and 0.352, *p* < 0.01, respectively; Table 3).

## 3. Discussion

Metabolomics is an advanced technology for rapid analysis of systems biology with high-throughput detection methods and data processing, and it has been widely used in early screening, diagnosis and prognosis of diseases, identification of new drug targets and monitoring of therapeutic effects [28,29]. Metabolites are the most downstream products of cell metabolism; hence, metabolomics analysis of these small-molecule components is conducive to understanding the changes in biological systems at the cellular level [29,30]. In recent years, metabolomics methods have been applied to investigate metabolites and study biomarkers in asthma patients [28,29,31,32,33]. However, there is a lack of research on SCIT based on single or mixed allergens as immune agents to treat AR, and there has not been any metabolomic analysis on their efficacy.

This study conducted a metabolomics analysis on serum samples from AR patients who had received SM-SCIT or DM-SCIT for up to 36 weeks. Metabolomics and multivariate analysis (Figure 3 and Figure 4, and Supplementary Figure S2) results showed that the downstream products of linoleic acid metabolism (i.e., 13-HODE, 9-HPODE, 5(S)-HETE, 8(S)-HETE, 11(S)-HETE, 15(S)-HETE and 11- dehydro-TXB2), which were associated with the AA pathway, decreased significantly, and the α-linolenic acid and EPA pathway downstream products 5-HEPE and 12-HEPE were significantly different. In addition, ω-6 polyunsaturated fatty acids (i.e., 4,7,10,13-docosatetraenoic acid and 7,10,13-eicosatrienoic acid) and ω-3 polyunsaturated fatty acids (i.e., 5,9,12-octadecatrienoic acid and 4,7,10,13,16,19-docosahexaenoic acid) also significantly decreased, but there was no significant difference between SM-SCIT and DM-SCIT groups. The results were consistent with VAS and RQLQ scores. Furthermore, the correlation analysis between the components in the SCIT process indicated that the components with similar carbon chain lengths had stronger correlations (Supplementary Figure S2). The changes of the above serum metabolic components (5(S)-HETE, 8(S)-HETE, 11(S)-HETE, 15(S)-HETE and 11-hydro TXB2) were correlated with the magnitude of RQLQ improvement, respectively. However, there was no significant difference in the overall metabolic components between patients treated with different methods. Comparing the changes in the content of metabolites in the two groups of AR patients, we found that the content of 11(S)-HETE in the SM-SCIT group decreased more than that in the DM-SCIT group.

AA and its downstream metabolites are key factors in inflammatory response [34,35]. Xie et al. collected serum samples from AR patients with sublingual immunotherapy (SLIT) and utilized the samples to obtain metabolomics profiling by applying UHPLC-MS, which found that AA decreased in the effective group, and they identified AA as one of the biomarkers that can reliably and accurately predict the efficacy of SLIT in AR patients [36]. When the respiratory epithelium is stimulated or immunomodulated, AA is oxidized and metabolized by LOX and GPX enzymes. LOX can be divided into 5-, 8-, 11-, 12- or 15-LOX according to the oxygenated position, and leading to oxidation reactions that are based on the catalysis of them, AA is metabolized into 5 (S)-, 8 (S)-, 11 (S)-, 12 (S)- and 15 (S)-HPETE [37]. GPX enzymes further metabolize HPETE into 5 (S)-, 12 (S)- and 15 (S)-HETE, respectively. HETEs were reportedly associated with promoting inflammation, whereby the respiratory infection activates HETEs, inducing inflammation [38]. In addition, higher concentrations of HETEs can activate peroxisome proliferator-activated receptors (PPARs), further promoting inflammation [39,40,41]. Moreover, studies have revealed that 15-HETE is positively correlated with AR and asthma [42,43], and we found that HETEs were significantly reduced in rhinitis patients after SCIT. Therefore, HETEs could not only be used as a potential target of inflammation during HDM SCIT in asthma patients [44], but also in rhinitis patients, and can explain the mechanism of this treatment.

11(S)-HETE is a downstream oxylipid of the AA/COX-1 pathway, which mainly produces by COX enzymes, and may also contribute to the production by LOX, CYP450 enzymes and non-enzymatic catalytic pathways [45]. According to reports, 11(S)-HETE, like other HETEs, has a positive correlation with inflammation. In addition, 11(S)-HETE is also a biomarker of coronary heart disease, coronary syndrome and cancer, but its biological function remains unclear [46,47,48]. Studies found that 11(S)-HETE stimulated endothelial cell proliferation, migration and angiogenesis, and then tumor growth and metastasis [48]. The current research on 11(S)-HETE is still superficial, but we found that the level of 11(S)-HETE in patients who received SM-SCIT decreased faster than those who received DM-SCIT, which may be due to its positive correlation with inflammation. Thus, we speculate that SM-SCIT can reduce the inflammation level in AR patients more effectively, and 11(S)-HETE can act as a biomarker to distinguish between these two SCIT.

The advantage of this study is that it is the first to analyze the long-term and longitudinal metabolic changes within AR patients treated with SM-SCIT and DM-SCIT. In the present study, HETE components were used as candidate biomarkers to monitor the treatment response related to SM- and DM-SCIT in AR patients, but not to indicate the severity or clinical effect of AR. Following SCIT treatment, the levels of AA and its downstream metabolic molecules (13-HODE, 9-HPODE, 5(S)-HETE, 8(S)-HETE, 11(S)-HETE, 15(S)-HETE and 11-hydro TXB2) decreased, but there was no significant difference between the two SCITs overall. Therefore, HETE components are potential biomarkers in SM-SCIT and DM-SCIT, and these metabolites may be used as new biological indicators to monitor the desensitization effect on HDM SCIT and to distinguish the two treatment schemes. 

There are some limitations to the study. First, we did not include a placebo arm. To avoid observer bias, we removed patients’ names and the date of examination, and blood samples were coded and analyzed randomly. Second, the short-term follow-up could be overcome through validation using patients with two types of SCIT treatment. As previously reported, the clinical effect is lost if sublingual immunotherapy is discontinued at two years [49], which suggests that longer observation periods of at least three years are required, as seen in the metabolic changes of allergic asthma patients with SCIT [44]. Lastly, future long-term prospective studies in bigger cohorts will allow for deeper analysis of the metabolic changes of AR and clarify their relationship with clinical effect.

Studies indicate that polyunsaturated fatty acids (PUFAs) and their metabolites can resolve inflammation, such as alpha-linolenic acid, linoleic acid and AA, but diet could affect the levels of these metabolites. Walnuts combined with physical activity reduced arachidonic acid-based oxylipin levels in the brain [50]. Supplementation with *C. butyricum* increased the concentrations of essential amino acids and flavor amino acids, as well as AA, docosahexaenoic acid (DHA), eicosapentaenoic acid (EPA) and total PUFAs in breast muscle [51]. Epigallocatechin gallate (EGCG) could induce enhanced lipid metabolism pathways, and the combination effect between EGCG and dietary restriction led to overactivation of linoleic acid and arachidonic acid oxidation pathways, significantly increasing the accumulation of pro-inflammatory lipid metabolites [52]. One of the major components in high-fat diets is the omega-6 PUFAs, called linoleic acid, which are metabolized to an array of eicosanoids and prostaglandins depending upon the enzymes in the pathway. Omega-3 fatty acids, such as α-linolenic acid (ALA), which are substrate competitors of linoleic acid and AA, were found to reduce LOX-mediated HETE and increase LOX-mediated HDHA in tissue and plasma after an ALA-rich diet [38]. However, PUFAs and their interactions in allergic disease are poorly understood, and further studies are necessary to understand the influence of diet.

## 4. Materials and Methods

### 4.1. Study Design and Population

A total of 219 serum samples were collected from 73 AR patients: 35 patients who received a *Der p* allergen preparation (single-species mite SCIT, SM-SCIT group) in 3 treatment periods (baseline (V0), the completion of initial treatment (V1) and the first stage of maintenance treatment (V2)), and 38 patients who received a mixed preparation of *Der p* and *Der f* (1:1) (double-species mite SCIT, DM-SCIT group) in 3 treatment periods (V0, V1, V2). The serum required no hemolysis, blood lipids and more than 50 µL for the consistency in metabolomic analysis. Visual analogue scale (VAS) and rhinoconjunctivitis quality of life questionnaire (RQLQ) were serially followed up at three periods. Of the patients, 68% were being treated with a drug for allergic rhinitis symptoms. Among them, 83.2% were taking oral H1-antihistamines, 24.2% intranasal corticosteroids and 17.8% had other treatment. Medications were not stopped before V1 was performed, but almost stopped drug treatment after V1. The study protocol was approved by the Ethics Committee of the First Affiliated Hospital of Guangzhou Medical University (ethics approval No. gyfyy-2016-73). Written informed consent was obtained from the parents of all study participants.

### 4.2. Inclusion and Exclusion Criteria 

Eligible patients were those with AR symptoms present when exposed to HDM. A positive skin prick test (SPT) response (skin wheal index ≥ 2) to *Der p* and *Der f*, and a specific IgE (sIgE) concentration > 0.7 IU/mL against *Der p*/*Der f* (ALLERG-O-LIQ system, Dr. Fooke Labs, Neuss, Germany) at screening were also required. 

Patients who had received subcutaneous or sublingual immunotherapy, or for whom epinephrine was contraindicated, were excluded from participating in the study. Other key exclusion criteria comprised asthma, irreversible airway damage, pregnancy, severe autoimmune disease, renal disease, chronic hepatic disease or lack of adherence. In addition, SCIT cases with missing serum samples during treatment at three time points were excluded.

### 4.3. Clinical Response 

VAS and RQLQ assessments of rhinitis symptoms at V0, V1 and V2 were completed by patients. Five specific clinical symptoms, including sneezing, runny, blocked or itchy nose and eye-related symptoms were assessed in overall VAS scores. Twenty-eight items in seven domains were recorded in RQLQs, including activity limitations, sleep problems, non-nose/eye-related symptoms, practical issues, nose-related symptoms, eye-related symptoms and emotional function [53].

### 4.4. Immunologic Response

#### 4.4.1. Skin Prick Testing 

We used a standard prick allergen kit (Aller-gopharm) (including *Der p*, *Der f* and Blot at V0 and V2) for the skin prick test (SPT), and calculated the mean wheal diameter (longest diameter plus shortest diameter perpendicular to it divided by 2) to determine the final wheal size. Wheals with a diameter exceeding 3 mm were considered positive. The following formula was used to calculate the skin wheal index (SI): SI = the mean wheal diameter of allergen/the mean wheal diameter of histamine. The size of SI was used to categorize SPT-positive response into four grades: grade 1 (SI < 0.5), grade 2 (0.5 ≤ SI < 1), grade 3 (1 ≤ SI < 2) and grade 4 (SI ≥ 2).

#### 4.4.2. Immunoglobulins 

An enzyme immune assay following the manufacturer’s instructions (Fooke Labs) was used to quantitatively determine the levels of specific IgE and IgG4 against *Der p*, *Der f* and Blot at V0, V1 and V2.

### 4.5. Sample Preparation

The supernatants (50 µL) of all serum samples were stored at −80 °C after being centrifuged at 800 g for 10 min before further use, and 5 μL of each sample was mixed as a quality control (QC) sample. The derivatization method was used to enhance the sensitivity and separation efficiency of fatty acid detection when analyzed through UHPLC-Q-TOF/MS (Agilent, Santa Clara, CA, USA). Serum samples were prepared using our previously developed approach and (2-aminoethyl) trimethylammonium chloride hydrochloride (cholamine) was selected as the derivatization reagent. The detailed information about fatty acids, internal standards and other reagents are shown in s-Appendix. Finally, 1 µL of the supernatant was injected directly into the UHPLC-Q-TOF/MS. The samples were injected in random order and a QC sample was injected every 7 samples.

### 4.6. UHPLC-Q-TOF-MS Analysis

The Agilent 1290 Infinity LC system (UHPLC, Santa Clara, CA, USA) was used to separated metabolites, which consisted of an autosampler, a thermostatically regulated column compartment and a binary pump with an Agilent Eclipse XDB-C18 column (2.1 × 100 mm, 1.8 μm, Santa Clara, CA, USA). Mass spectrometry was conducted on an Agilent 6550 UHD (Santa Clara, CA, USA) accurate mass Q-TOF/MS system with a dual-jet stream electrospray ion source (dual AJS ESI). The detailed elution procedure and MS parameters are shown in s-Appendix.

### 4.7. Data Preprocessing and Statistical Analysis

Raw LC-MS data from the SM-SCIT and DM-SCIT groups were acquired and processed using Agilent MassHunter Qualitative Analysis B.06.00 software (Agilent Technology, Santa Clara, CA, USA). Metabolites were identified using standards, MS/MS spectra, and the Lipid Maps (http://www.lipidmaps.org/, accessed on 20 March 2021) and METLIN (https://metlin.scripps.edu/index.php, accessed on 20 March 2021) metabolite databases. Clinical and metabolomic data were processed using IBM SPSS statistics 20 and GraphPad Prism 5.0 statistical software (GraphPad, Inc., La Jolla, CA, USA). Qualitative data were reported as a percentage showing the proportion of positive results and analyzed via the chi-squared test. Non-parametric quantitative data were represented by the median (interquartile range). The Wilcoxon signed-rank test for two samples and the one-way repeated measures ANOVA for multiple samples were performed for within-group comparisons. The Mann–Whitney U test was performed for between-group comparisons. As a consequence, using the identified metabolites as variables, the principal component analysis (PCA) and OPLS-DA were performed to assess the relationship between V0 and V2 groups in DM-SCIT or SM-SCIT groups using SIMCA-P software (version 13.0; Umetrics, Umea, Sweden). The relationship between covariance and correlation within OPLS-DA was visualized by calculation of variable importance in projection (VIP) values. Furthermore, Student’s t-test was used to measure the significance of metabolites in groups. A correlation heat map was used to describe the relationship between changes (Δ: post-treatment minus pre-treatment) in VAS and RQLQ scores and metabolites. A *p*-value < 0.05 was considered significant.

## 5. Conclusions

In this study, AR patients that had received SM-SCIT or DM-SCIT were monitored dynamically, and the changes in the content of metabolic components in patients were assessed by derivatization with UHPLC-Q-TOF/MS. The results confirmed that both therapies had therapeutic efficacy in rhinitis patients, which was proven by the decrease in inflammation-related AA pathway metabolites (13-HODE, 9-HPODE, 5-HETE, 8-HETE, 11-HETE, 15-HETE and 11-hydro TXB2). Moreover, although there was no significant difference between the effects of the two therapeutic schemes, it was found that 11(S)-HETE, an inflammation-related metabolite, may be a potential biomarker for distinguishing them.

## Figures and Tables

**Table 1:** Baseline characteristics and demographics.

Characteristics	SM-SCIT	DM-SCIT	*p*
No.	35	38	-
Sex (Male), No. (%)	22 (62.9)	27 (71.1)	0.456
Age (years), median (IQR)	11.00 (2.5)	10.50 (6.3)	0.881
<18 years, No. (%)	32 (91.4)	31 (81.6)	0.378
AR combined with allergic asthma, No. (%)	1 (2.9)	2 (5.3)	1.000
Atopic family history ^a^, No. (%)	20 (57.1)	19(50.0)	0.541
Score, median (IQR)			
Overall VAS	24.60 (8.6)	25.40 (8.3)	0.782
Sneezing	5.10 (2.5)	5.00 (3.8)	0.760
Runny nose	5.50 (2.7)	5.00 (3.0)	0.650
Blocked nose	5.10 (2.9)	5.00 (3.0)	0.130
Itchy nose	5.10 (2.4)	4.80 (2.7)	0.800
Eye symptoms	3.80 (2.8)	3.60 (2.7)	0.810
Overall RQLQ	54.00 (27.6)	51.50 (30.5)	0.493
Activity limitations	6.00 (3.7)	6.00 (3.8)	0.530
Sleep problems	5.60 (3.9)	4.00 (4.5)	0.680
Non-nose/eye symptoms	11.90 (7.6)	12.50 (8.0)	0.760
Practical problem	7.90 (3.8)	7.00 (3.0)	0.510
Nose symptoms	10.20 (5.1)	11.50 (4.3)	0.210
Eye symptoms	4.00 (4.0)	5.40 (4.7)	0.530
Emotional function	7.30 (5.9)	7.20 (5.4)	0.910
Classification of severity, No. (%)			
Mild intermittent	12 (34.3)	13 (34.2)	0.995
Mild persistent	8 (22.9)	9 (23.7)	0.933
Moderate/severe intermittent	11 (31.4)	10 (26.3)	0.630
Moderate/severe persistent	4 (11.4)	6 (15.8)	0.841
SPT, SI/No.(%)			
*Der p*, Median (IQR)	3.00 (1.0)	3.00 (1.0)	0.450
*Der f*, Median (IQR)	3.00 (1.0)	3.00 (0.8)	0.077
*Blo t*, Median (IQR)	2.00 (2.5)	0.00 (2.0)	0.991
Animal allergens, No. (%)	8 (22.9)	12 (31.6)	0.404
Grass pollens, No. (%)	1 (2.9)	5 (13.2)	0.240
Mold allergens, No. (%)	0 (0.0)	3 (7.9)	0.241
sIgE (IU/mL), median (IQR)			
*Der p*-sIgE	15.81 (35.1)	26.27(58.1)	0.306
*Der f*-sIgE	18.87 (26.8)	22.52 (36.4)	0.456
Blo t-sIgE	0.20 (0.2)	0.25 (0.2)	0.716
sIgG4 (U/mL), median (IQR)			
*Der p*-sIgG4	34.17 (7.5)	35.17 (8.3)	0.134
*Der f*-sIgG4	164.89 (201.4)	127.91 (321.3)	0.216
Blo t-sIgG4	41.78 (12.4)	36.82 (12.5)	0.298

^a^: These included AR, asthma, eczema, atopic dermatitis, food allergy and so on. There was 1 missing date in each group. *Blo t*: Blomia tropicalis; sIgE: specific IgE; sIgG4: specific IgG4; IQR: Interquartile range.

**Table 2:** Comparison of inflammation-related metabolite content between patients during pre-treatment (V0) and the first stage of the maintenance phase (V2) (√: the metabolite is mainly metabolized through this metabolic pathway).

Compound Name	Formula	Metabolized from	Enzyme	Pathways			*p*-Value	
				Linoleic Acid Metabolism	*α*-Linolenic Acid Metabolism	AA Metabolism	DM	SM
15(S)-HETE	C_20_H_32_O_3_	AA	15-LOX,GPX4			√	0.0004 ***	0.005 **
11(S)-HETE	C_20_H_32_O_3_	AA	11-LOX,GPX4			√	0.001 **	0.053
12(S)-HETE	C_20_H_32_O_3_	AA	12-LOX,GPX4			√	0.313	0.422
8(S)-HETE	C_20_H_32_O_3_	AA	8-LOX,GPX4			√	0.002 **	0.052
5(S)-HETE	C_20_H_32_O_3_	AA	5-LOX,GPX4			√	0.001 **	0.014 *
13(S)-HPODE	C_18_H_32_O_4_	Linoleic acid	15-LOX	√			0.701	0.265
9(S)-HPODE	C_18_H_32_O_4_	Linoleic acid	9-LOX	√			0.519	0.025 *
15(S)-HEPE	C_20_H_30_O_3_	EPA	15-LOX,GPX4			√	0.617	0.154
12(S)-HEPE	C_20_H_30_O_3_	EPA	12-LOX,GPX4			√	0.027 *	0.018 *
5(S)-HEPE	C_20_H_30_O_3_	EPA	5-LOX,GPX4			√	0.009**	0.057
13-HODE	C_18_H_32_O_3_	Linoleic acid	15-LOX	√			0.004 **	0.020 *
AA	C_20_H_32_O_2_	Linoleic acid	Delta6-desaturase	√			0.002 **	0.219
13(S)-HOTrE	C_18_H_30_O_3_	Linoleic acid	13-LOX	√			0.491	0.069
TXB2	C_20_H_34_O_6_	AA	COX			√	0.607	0.225
12(S)-HHTrE	C_17_H_28_O_3_	AA	COX			√	0.597	0.768
11-dehydro TXB2	C_20_H_32_O_6_	AA	COX			√	0.882	0.518
EPA	C_20_H_30_O_2_	α-Linolenic acid	Delta6-desaturase		√		0.032 *	0.207
α-Linolenic acid	C_18_H_30_O_2_	-			√		0.0004 ***	0.302

HETE: hydroxyeicosatetraenoic acid; HEPE: hydroxyeicosapentaenoic acid; HPODE: hydroperoxylinoleic acid; HODE: hydroxyoctadecadienoic acid; HOTrE: hydroxyoctadecatrienoic acid; TXB2: thromboxane B2; HHTrE: hydroxyheptadecatrienoic acid; EPA: eicosapentaenoic acid; GPx: glutathione peroxidase; LOX: lipoxygenase; COX: cyclooxygenase. *, *p* < 0.05; **, *p* < 0.01; ***, *p* < 0.001.

**Table 3:** Correlation between RQLQ improvement and change in metabolites’ concentration.

Variables (∆)	∆ Overall RQLQ	
	*r*	*p*
15(S)-HETE	0.424	0.0002 ***
11(S)-HETE	0.418	0.0002 ***
11-dehydro TXB2	0.374	0.0011 **
5(S)-HETE	0.363	0.0016 **
8(S)-HETE	0.360	0.0017 **
13-HODE or isomer	0.353	0.0021 **

∆, post-treatment (V2) minus pre-treatment (V0); **, *p* < 0.01; ***, *p* < 0.001.

## Data Availability

The data presented in this study are available in supplementary material.

## Supplementary Materials

The following are available online at https://www.mdpi.com/article/10.3390/metabo11090613/s1, s-Appendix 1: Chemicals and materials, s-Appendix 2: Sample preparation, s-Appendix 3: UHPLC-Q-TOF/MS analysis, Figure S1: Correlation heat map of metabolites in patients during DM-SCIT or SM-SCIT, Figure S2: Score plots of PCA-X and OPLS-DA models between V0 and V2 groups in DM-SCIT or SM-SCIT groups, Table S1: Comparison of the characteristics of protocol groups and withdrawal groups, Table S2: Metabolites identified in serum using UHPLC-Q-TOF/MS analysis, Table S3: Correlation between symptoms’ improvement and change in metabolites’ concentration.
