# Supplementary material for: Metabolomics Reveals Process of Allergic Rhinitis Patients with Single- and Double-Species Mite Subcutaneous Immunotherapy
Source: Metabolites. 2021 Sep 9;11(9):613. doi: 10.3390/metabo11090613 (PMC8471092; doi:10.3390/metabo11090613)
Supplement: Supplementary file 1 [file metabolites-11-00613-s001.zip › metabolites-1257849-supplementary.pdf]

Metabolomics reveals process of allergic rhinitis patients with single-and double-species mite subcutaneous immunotherapy

Peiyan Zheng[1#], Guanyu Yan[1#], Yida Zhang[1#], Huimin Huang[1], Wenting Luo[1], Mingshan Xue[1], Na Li[2], Jian-Lin Wu [2*], Baoqing Sun[1*]

# These authors contributed equally to the study.

* Correspondence: Baoqing Sun, The First Affiliated Hospital of Guangzhou Medical University, Guangzhou, sunbaoqing@vip.163.com; Jian-Lin Wu, Macau University of Science and Technology, Macao, jlwu@must.edu.mo

SUPPLEMENTAL MATERIAL

Contents:

s-Appendix 1. Chemicals and materials
s-Appendix 2. Sample preparation
s-Appendix 3. UHPLC-Q-TOF/MS analysis
Figure S1. Correlation heat map of VAS，RQLQ scores and metabolites in patients during DM-SCIT or SM-SCIT.
Figure S2. Score plots of PCA-X (a, c) and OPLS-DA (b, d) models between V0 and V2 groups in DM-SCIT (a, b) or SM-SCIT (c, d). (Green: V0 group; Red: V2 group).
Table S1: Compare the characteristics of protocol groups and withdrawal groups
Table S2: Metabolites identified in serum using UHPLC-Q-TOF/MS analysis
Table S3: Correlation between symptoms improvement and change in metabolites concentration

s-Appendix 1. Chemicals and materials

Fatty acid standards, including 5(S)-, 8(S)-, 11(S)-, 12(S)-, and 15(S)-HETEs, 5(S)-, 12(S)-, and 15(S)-HPETEs, 9(S)- and 13(S)-HPODEs, 5(S)-, 12(S)-, and 15(S)- hydroxyeicosapentaenoic acids (HEPEs), 13(S)-hydroxyoctadecatrienoic acid (HOTrE); Prostaglandin A2 (PGA2), PGB2, PGD2, PGE2, PGJ2, PGE1, PGF1α, TXB2, 12(S)-hydroxyheptadecatrienoic acid (HHTrE), and 13,14-dihydro-15-keto PGF2α, were purchased from Cayman Chemical (Ann Arbor, MI, USA). The isotope-labeled reagents 12(S)-HETE-d8 and PGD2-d4 were used as internal standards. The other standards hexanoic acid, octanoic acid, decanoic acid, dodecanoic acid, myristic acid, stearic acid, heptadecanoic acid, docosahexaenoic acid, eicosapentaenoic acid, docosapentaenoic acid, oleic acid, chenodeoxycholic acid, deoxycholic acid, and isodeoxycholic acid were obtained from Sigma-Aldrich (St. Louis, MO, USA) and J&K Scientific (Beijing, China).

(2-aminoethyl) trimethylammonium chloride hydrochloride (cholamine), 1-hydroxybenzotriazole hydrate (HOBt), O-(7-azabenzotriazol-1-yl)-N,N,N′,N′-tetramethyluronium hexafluorophosphate (HATU) and triethylamine (TEA) were purchased from Sigma-Aldrich. Acetonitrile (ACN, HPLC grade) and methanol (MeOH, HPLC grade) were obtained from Anaqua Chemicals Supply Inc., Ltd. (Houston, TA, USA). Deionized water was prepared using a Millipore water purification system (Millipore Corp, USA). Mass spectrometry (MS) grade formic acid and other chemical reagents were provided by Sigma-Aldrich.

s-Appendix 2. Sample preparation

The supernatants (50 μL) of all serum samples were stored at -80 °C after centrifuged at 800 g for 10 min before further use. 5μl of each samples were mixed as QC sample. Metabolites were analyzed using our previously developed UHPLC-Q-TOF/MS approach [27]. Briefly, 50 μL of each serum sample and 200 μL of cold ethyl acetate were mixed and centrifuged at 14500 g for 5 min (4 °C), repeated the above extraction three times. Then the combined supernatants were dried under nitrogen stream. 5 μL of 20 mM HOBt in dimethyl sulfoxide (DMSO), 10 μL of 100 mM cholamine in DMSO containing 200 mM TEA, and 5 μL of 20 mM HATU in DMSO were sequentially mixed with the residues of each 50 μL aliquot of serum sample, and then incubated for 1 min at room temperature. Next, 30 μL of acetonitrile was added to make up a final volume of 50 μL, then centrifuged at 14500 g for 10 min at 4 °C. Finally, 1 μL of the supernatant was injected directly into the UHPLC-Q-TOF/MS. The samples were injected in random order and QC sample was injected every 7 samples.

s-Appendix 3. UHPLC-Q-TOF/MS analysis

Agilent 1290 Infinity LC system (UHPLC, Santa Clara, CA, USA) were used to separated metabolites, which using an consisting of an autosampler, a thermostatically regulated column compartment, and a binary pump with an Agilent Eclipse XDB-C18 column (2.1 × 100 mm, 1.8 μm). The column temperature was maintained at 40 °C and the autosampler was set at 4 °C. Mobile phase A and B were 0.1 % formic acid-containing water and 0.1 % formic acid-containing acetonitrile, respectively, and the gradient was set as follows: 0-1 min, 15-23 % B; 1-

8 min, 23-33 % B; 8-8.5 min, 33-35 % B; 8.5-15.5 min, 35-47 % B; 15.5-16 min, 47-50 % B; 16-23 min, 50-85 % B; 23-25 min, 85-95 % B; 25-28.9 min, 95 % B. The injection volume was 1 μL and the flow rate was 0.3 mL/min.

Mass spectrometry was conducted on an Agilent 6550 UHD accurate mass Q-TOF/MS system with a dual jet stream electrospray ion source (dual AJS ESI). The instrument was operated in positive full scan mode with the following MS parameters: dry gas temperature, 250 °C; dry gas flow, 15 L/min; sheath gas temperature, 300 °C; sheath gas flow, 11 L/min; nebulizer pressure, 20 psi; capillary voltage, 5000 V; and nozzle voltage, 500 V. Mass spectra were recorded between 200 and 1000 *m*/*z*. Accurate mass measurements were obtained by using a low flow of TOF reference mixture (reference masses: *m*/*z* 322.0481, 622.0289, 922.0098), containing internal reference masses at *m*/*z* 922.0098 ($C_{18}H_{18}F_{24}N_3O_6P_3$).

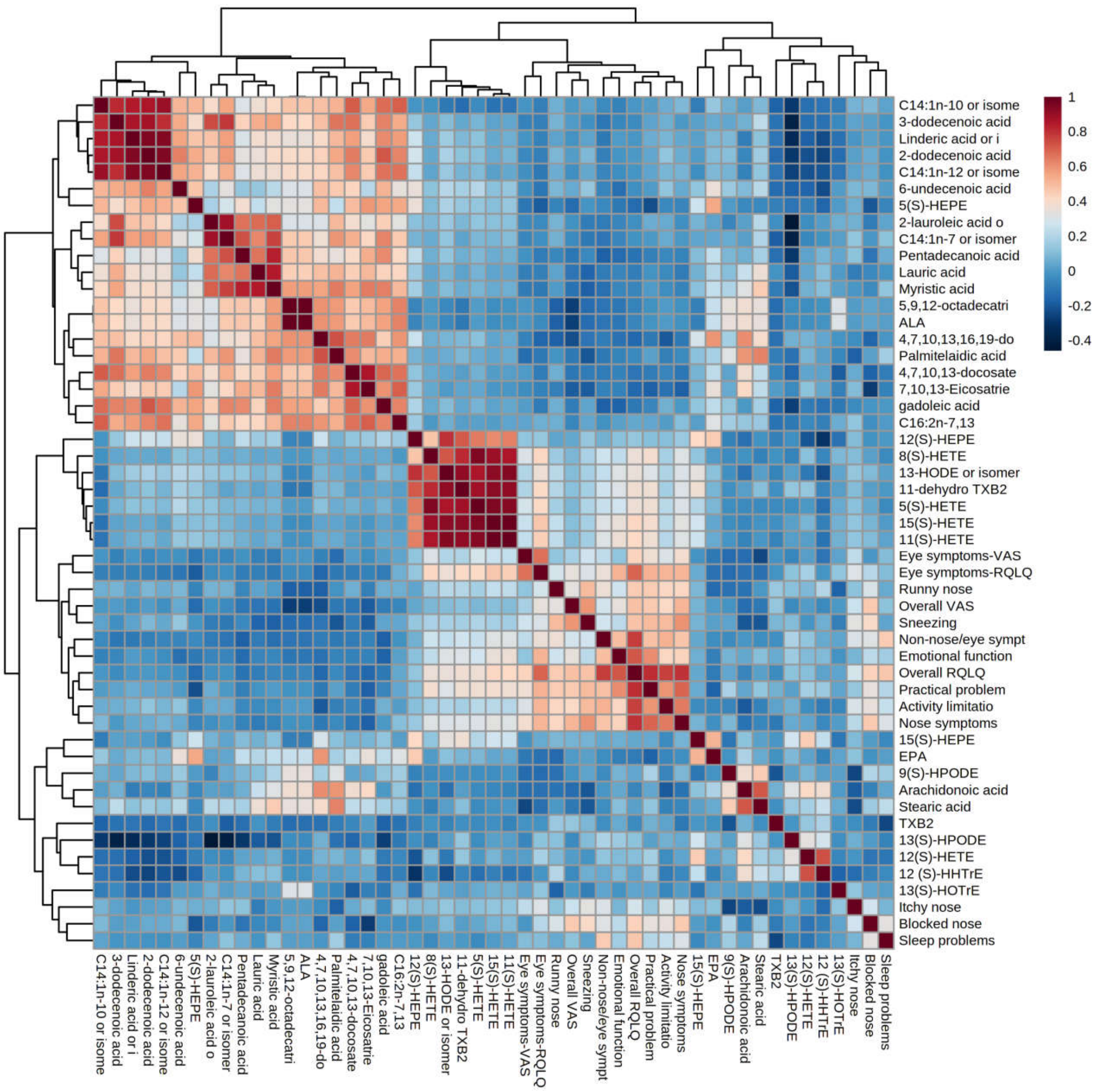

Figure S1. Correlation heat map of VAS，RQLQ scores and metabolites in patients during DM-SCIT or SM-SCIT. Red cells represent higher levels of the specific metabolite in that sample, whereas blue cells rep-resent lower levels.

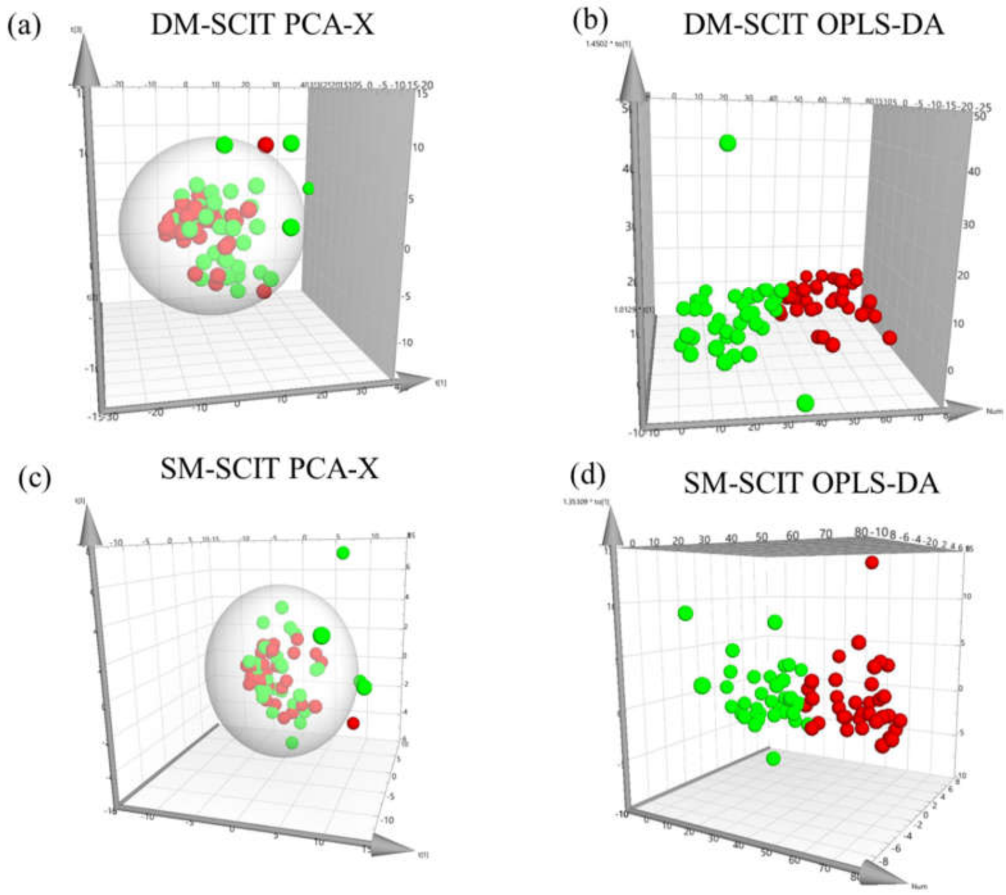

Figure S2. Score plots of PCA-X (a, c) and OPLS-DA (b, d) models between V0 and V2 groups in DM-SCIT (a, b) or SM-SCIT (c, d). (Green: V0 group; Red: V2 group).

Table S1. Compare the characteristics of the protocol groups and withdrawal groups

Characteristics	Protocol groups			Withdrawal groups			Protocol vs withdrawal	
	SM-SCIT	DM-SCIT	*P*	SM-SCIT	DM-SCIT	*P*	SM-SCIT *P*	DM-SCIT *P*
No.	35	38	-	28	24	-	-	-
Sex (Male), No. (%)	22 (62.9)	27 (71.1)	0.456	22 (78.6)	19 (79.2)	0.958	0.177	0.477
Age (years), median (IQR)	11.00 (2.5)	10.50 (6.3)	0.881	12（15.3）	11（6.3）	0.285	0.236	0.706
< 18 years, No. (%)	32 (91.4)	31 (81.6)	0.378	21 (75.0)	21 (87.5)	0.431	0.154	0.793
AR combined with allergic asthma, No. (%)	1 (2.9)	2 (5.3)	1.000	3 (10.7)	0 (0.0)	0.240	0.315	0.518
Atopic family history a, No. (%)	20 (57.1)	19(50.0)	0.541	11 (39.3)	13 (54.2)	0.283	0.159	0.749
Overall VAS score, median (IQR)	24.64 (8.6)	25.44 (8.3)	0.782	21.5 (13.8)	20 (13.8)	0.701	0.074	0.055
Overall RQLQ score, median (IQR)	54.00 (27.6)	51.50 (30.5)	0.493	43.5 (33.5)	39.5 (39.5)	0.708	0.397	0.174
SPT (SI), *Der p*, Median (IQR)	3.00 (1.0)	3.00 (1.0)	0.450	3.00 (2.0)	3.00 (1.0)	0.794	0.770	0.521
SPT (SI), *Der f*, Median (IQR)	3.00 (1.0)	3.00 (0.8)	0.077	2.00 (1.0)	3.00 (1.0)	0.544	0.679	0.103
SPT (SI), *Blo t*, Median (IQR)	2.00 (2.5)	0.00 (2.0)	0.991	2.00 (1.0)	2.00 (1.3)	0.124	0.268	0.715
Der p and *Der f* sIgE (IU/mL), median (IQR)	8.59 (23.4)	24.00 (30.4)	0.779	23.46 (23.1)	20.75 (29.1)	0.557	0.946	0.761

sIgE: specific IgE; *Der p* and *Der f* sIgE: *Der p* and *Der f* mixed allergen sIgE test; IQR: Inter Quartile Range.

Table S2. Metabolites identified in serum using UHPLC-Q-TOF/MS analysis

No.	Compound Name	Formula	Derivatization Formula	Precursor ions $[M]^+$	RT (min)	MS/MS	*P* Value and VIP value	
							DM	SM
1	Arachidonoic acid	$C_{20}H_{32}O_2$	$C_{25}H_{45}N_2O$	389.3526	19.3	330.27, 302.24, 245.22, 218.15, 192.13, 166.12, 152.10, 98.06, 60.08	0.002**,1.5	0.219, -

No.	Compound Name	Formula	Derivatization Formula	Precursor ions $[M]^+$	RT (min)	MS/MS	*P* Value and VIP value	
							DM	SM
2	13(S)-HPODE	$C_{18}H_{32}O_4$	$C_{23}H_{45}N_2O_3$	397.343	12.9	320.25, 292.26, 250.18, 222.18, 194.15, 180.13, 166.12, 123.11, 98.06, 60.08	0.701, -	0.265, -
3	9(S)-HPODE	$C_{18}H_{32}O_4$	$C_{23}H_{45}N_2O_3$	397.343	13.1	320.01, 292.26, 264.19, 250.18, 208.13, 182.15, 140.91, 122.09, 104.84, 60.08	0.519, -	0.025*, 1.5
4	15(S)-HETE	$C_{20}H_{32}O_3$	$C_{25}H_{45}N_2O_2$	405.3481	14.04	328.26, 300.23, 243.21, 215.17, 173.13, 145.10, 131.08, 117.07, 98.06, 60.08	0.0004***, -	0.005**, 1.3
5	11(S)-HETE	$C_{20}H_{32}O_3$	$C_{25}H_{45}N_2O_2$	405.3481	14.7	328.26, 243.21, 214.91, 152.10, 98.06, 60.08	0.001**, -	0.053, 1.2
6	12(S)-HETE	$C_{20}H_{32}O_3$	$C_{25}H_{45}N_2O_2$	405.3481	14.9	328.26, 300.23, 243.21, 217.14, 138.09, 98.06	0.313, -	0.422, -
7	8(S)-HETE	$C_{20}H_{32}O_3$	$C_{25}H_{45}N_2O_2$	405.3481	15.3	346.27, 328.26, 243.21, 219.15, 192.13, 152.10, 138.09, 98.06, 60.08	0.002**, -	0.052, 1.2
8	5(S)-HETE	$C_{20}H_{32}O_3$	$C_{25}H_{45}N_2O_2$	405.3481	16.1	346.27, 328.26, 286.08, 243.21, 201.16, 178.12, 131.08, 98.06, 60.08	0.001**, -	0.014*, 1.1
9	15(S)-HEPE	$C_{20}H_{30}O_3$	$C_{25}H_{43}N_2O_2$	403.3325	13.2	326.24, 289.98, 262.13, 244.17, 143.08, 98.06, 60.08	0.617, -	0.154, 1.1

No.	Compound Name	Formula	Derivatization Formula	Precursor ions $[M]^+$	RT (min)	MS/MS	*P* Value and VIP value	
							DM	SM
10	12(S)-HEPE	$C_{20}H_{30}O_3$	$C_{25}H_{43}N_2O_2$	403.3325	14.3	326.24, 284.21, 241.19, 218.15, 192.13, 150.12	0.027*, -	0.018*, 1.5
11	5(S)-HEPE	$C_{20}H_{30}O_3$	$C_{25}H_{43}N_2O_2$	403.3325	15.9	326.24, 298.87, 243.09, 178.12, 147.06, 121.06, 105.07, 69.03	0.009**, -	0.057, 1.4
12	13(S)-HOTrE	$C_{18}H_{30}O_3$	$C_{23}H_{43}N_2O_2$	379.3325	13.1	320.25, 302.24, 285.22, 259.20, 217.19, 189.16, 121.10, 93.06, 60.08	0.491, -	0.069, 1.0
15	TXB2	$C_{20}H_{34}O_6$	$C_{25}H_{47}N_2O_5$	455.3485	4.6	378.26, 360.25, 342.24, 324.22, 306.24, 288.2324, 260.16, 194.11, 152.10, 98.06, 60.07	0.607, -	0.225, -
16	12 (S)-HHTrE	$C_{17}H_{28}O_3$	$C_{22}H_{41}N_2O_2$	365.3168	10.9	306.24, 288.23, 260.20, 203.17, 138.09, 119.08, 98.06, 60.08	0.597, -	0.768, -
17	11-dehydro TXB2	$C_{20}H_{32}O_6$	$C_{25}H_{45}N_2O_5$	453.3325	5.7	394.16, 376.25, 360.25, 332.26, 288.23, 229.19, 203.18, 138.09, 98.06, 69.63	0.882, -	0.518, 1.4
18	EPA	$C_{20}H_{30}O_2$	$C_{25}H_{43}N_2O$	387.3375	18.3	328.2632, 246.1854, 220.1693, 192.1389, 166.1233, 152.1066, 138.0918, 119.0854, 98.0606, 60.0807	0.032*, -	0.207, 1.3

No.	Compound Name	Formula	Derivatization Formula	Precursor ions $[M]^+$	RT (min)	MS/MS	*P* Value and VIP value	
							DM	SM
19	ALA	$C_{18}H_{30}O_2$	$C_{23}H_{43}N_2O$	363.3375	17.9	304.2642, 276.2342, 262.2171, 248.2018, 222.1858, 208.1707, 194.1547, 168.1391, 154.1235, 121.1020,	0.0004***, 2.1	0.302, -
20	Ursodeoxycholic acid	$C_{24}H_{40}O_4$	$C_{29}H_{53}N_2O_3$	477.4039	6.3	418.33, 330.16, 295.24, 265.04, 232.47, 232.47, 190.91, 60.08	0.809, -	0.471, -
21	Chenodeoxycholic acid	$C_{24}H_{40}O_4$	$C_{29}H_{53}N_2O_3$	477.4039	8.2	418.33, 397.73, 366.68, 335.56, 280.21, 239.15, 202.16, 123.08, 60.08	0.326, -	0.781, -
22	Deoxycholic acid	$C_{24}H_{40}O_4$	$C_{29}H_{53}N_2O_3$	477.4039	10.9	418.33, 402.26, 372.57, 328.36, 255.13, 213.16, 161.13, 128.10, 60.08	<0.001***, -	0.857, -
23	Isodeoxycholic acid	$C_{24}H_{40}O_4$	$C_{29}H_{53}N_2O_3$	477.4039	11.4	418.33, 400.32, 351.19, 300.22, 268.36, 161.13, 121.05, 60.08	0.00087***, -	0.01*, -
24	Myristic acid	$C_{14}H_{28}O_2$	$C_{19}H_{41}N_2O$	313.3207	17.1	289.98, 273.28, 254.24, 220.16, 138.13, 124.08	0.004**,1.9	0.174,1.2
25	Palmitelaidic acid	$C_{16}H_{30}O_2$	$C_{21}H_{43}N_2O$	339.3373	18.3	280.26, 262.25, 236.23, 196.20, 154.12, 126.09, 98.06, 60.08	0.0009***, 2.8	0.062, 1.2
26	Pentadecanoic acid	$C_{15}H_{30}O_2$	$C_{20}H_{43}N_2O$	327.3365	18.6	268.26, 210.18, 151.90, 109.10, 85.10, 60.08	0.037*, -	0.201, 1.3

No.	Compound Name	Formula	Derivatization Formula	Precursor ions $[M]^+$	RT (min)	MS/MS	*P* Value and VIP value	
							DM	SM
27	9,11-octadecadienoic acid	$C_{18}H_{32}O_2$	$C_{23}H_{45}N_2O$	365.3539	19	306.2792, 264.2326, 236.2010, 208.1702, 182.1544, 154.1232, 98.0608, 81.0705, 60.0815	0.502, -	0.909, -
28	Palmitic acid	$C_{16}H_{32}O_2$	$C_{21}H_{45}N_2O$	341.3541	19.6	282.27, 224.20, 210.18, 196.17, 182.15, 154.12, 123.11, 98.06, 57.07	0.313, -	0.201, -
29	Oleic acid	$C_{18}H_{34}O_2$	$C_{23}H_{47}N_2O$	367.3703	20.1	308.29, 266.24, 238.21, 210.18, 182.15, 154.12, 112.07, 98.06, 69.07	0.265, -	0.883, -
30	Stearic acid	$C_{18}H_{36}O_2$	$C_{23}H_{49}N_2O$	369.385	21.3	310.31, 266.24, 252.23, 238.21, 210.18, 182.15, 154.12, 128.1 98.06, 57.07	0.045*, 2.2	0.394, 1.2
31	C14:1n-7 or isomer	$C_{14}H_{26}O_2$	$C_{19}H_{39}N_2O$	311.3056	14.7	252.2335, 181.1586, 162.0915, 126.0929, 98.0613, 85.0546	0.022*, -	0.238, -
32	C14:1n-10 or isomer	$C_{14}H_{26}O_2$	$C_{19}H_{39}N_2O$	311.3056	15.2	252.2325, 229.9245, 184.2065, 152.1465, 126.0935, 98.0615	0.014*, -	0.844, -
33	C14:1n-12 or isomer	$C_{14}H_{26}O_2$	$C_{19}H_{39}N_2O$	311.3056	16.3	252.2332, 208.2079, 185.1052, 140.1077, 98.0602, 69.0700	0.017*, -	0.492, -

No.	Compound Name	Formula	Derivatization Formula	Precursor ions $[M]^+$	RT (min)	MS/MS	*P* Value and VIP value	
							DM	SM
34	C16:2n-7,13	$C_{16}H_{28}O_2$	$C_{21}H_{41}N_2O$	337.3217	16.6	278.2480, 239.0355, 210.1847, 166.1225, 147.1351, 121.1016, 98.0608, 60.0815	0.00005***, -	0.265, -
35	6-undecenoic acid	$C_{11}H_{20}O_2$	$C_{16}H_{33}N_2O$	269.259	8.8	210.1857, 196.1094, 170.0838, 126.0932, 98.0605, 69.0701	0.034*, -	0.394, -
36	2-lauroleic acid or isomer	$C_{12}H_{22}O_2$	$C_{17}H_{35}N_2O$	283.2742	10.9	224.2025, 179.9379, 98.0613, 70.0736	0.019*, -	<0.001***,1.4
37	3-dodecenoic acid or isomer	$C_{12}H_{22}O_2$	$C_{17}H_{35}N_2O$	283.2742	11.3	224.2015, 112.0917, 98.0598, 69.0706	0.045*, -	0.342, -
38	2-dodecenoic acid or isomer	$C_{12}H_{22}O_2$	$C_{17}H_{35}N_2O$	283.2742	12	224.2013, 138.8020, 98.0628	0.027*, -	0.635, -
39	Linderic acid or isomer	$C_{12}H_{22}O_2$	$C_{17}H_{35}N_2O$	283.2742	12.4	224.2007, 124.1134, 98.0598	0.034*, -	1, -
40	12-hydroxy-8,10-octadecadienoic acid or isomer	$C_{18}H_{32}O_3$	$C_{23}H_{45}N_2O_2$	381.3469	13.1	304.2645, 177.1291, 163.1488, 149.0247, 124.1137, 98.0614	0.25, -	0.125, -
41	13-HODE or isomer	$C_{18}H_{32}O_3$	$C_{23}H_{45}N_2O_2$	381.3469	13.6	304.2644, 152.1082, 98.0630	0.004**, -	0.020*, 1.3
42	5,9,12-octadecatrienoic acid or isomer	$C_{18}H_{30}O_2$	$C_{23}H_{43}N_2O$	363.3376	17.9	304.2634, 262.2166, 236.2009, 208.1697, 182.1540, 154.1228, 140.1077, 112.0759, 98.0605, 60.0814	0.0004***, 2.1	0.334, -

No.	Compound Name	Formula	Derivatization Formula	Precursor ions $[M]^+$	RT (min)	MS/MS	*P* Value and VIP value	
							DM	SM
43	4,7,10,13,16,19-docosa-hexaenoic acid	$C_{22}H_{32}O_2$	$C_{27}H_{45}N_2O$	413.3523	19.3	354.2787, 246.1852, 218.1547, 192.1382, 166.1228, 152.1072, 138.0919, 119.0859, 98.0604, 60.0814	0.0001***,1.7	0.036*, 1.3
44	C20:2n-5, 11	$C_{20}H_{36}O_2$	$C_{25}H_{49}N_2O$	393.3833	20.7	334.3107, 278.2476, 250.2169, 224.2021, 196.1707, 168.1393, 140.1080, 126.0925, 98.0604, 60.0815	0.394, -	0.318, -
45	15-methyl palmitic acid	$C_{17}H_{34}O_2$	$C_{22}H_{47}N_2O$	355.3676	20.6	296.2947, 252.2314, 224.2019, 196.1701, 168.1394, 140.1080, 98.0604, 57.0705	0.107, -	0.523, -
46	Gadoleic acid	$C_{20}H_{38}O_2$	$C_{25}H_{51}N_2O$	395.3991	21.7	336.3261, 294.2799, 252.2317, 224.2027, 196.1716, 168.1385, 140.1084, 98.0605, 60.0815	0.036*, -	0.623, -
47	Lauric acid	$C_{12}H_{24}O_2$	$C_{17}H_{37}N_2O$	285.2904	13.2	226.2169, 194.7862, 170.8422, 126.0916, 98.0605, 57.0705	0.013*,1.2	0.471, -
48	4,7,10,13-docosatetraenoic acid	$C_{22}H_{36}O_2$	$C_{27}H_{49}N_2O$	417.3834	20.6	358.3101, 330.2777, 297.2573, 274.2161, 208.1696, 180.1388,	0.007**, -	0.128, -

No.	Compound Name	Formula	Derivatization Formula	Precursor ions $[M]^+$	RT (min)	MS/MS	*P* Value and VIP value	
							DM	SM
						152.1070, 98.0605, 60.0808		
49	7,10,13-Eicosatrienoic acid	$C_{20}H_{34}O_2$	$C_{25}H_{47}N_2O$	391.3676	19.9	332.2946, 276.2320, 262.2164, 248.2009, 187.1484, 147.1172, 121.1014, 98.0604	0.0005***, -	0.062, -
50	Heptanoic acid	$C_7H_{14}O_2$	$C_{12}H_{27}N_2O$	215.2123	3.2	157.14, 113.09, 85.10, 60.08	0.232, -	0.128, -
51	Hexanoic acid isomer	$C_6H_{12}O_2$	$C_{11}H_{25}N_2O$	201.1966	1.7	142.13, 114.11, 99.09, 72.08	0.265, -	0.67, -
52	Hexanoic acid	$C_6H_{12}O_2$	$C_{11}H_{25}N_2O$	201.1966	1.9	142.13, 114.11, 99.09, 72.08	0.528, -	0.154, -
53	Octanoic acid isomer	$C_8H_{16}O_2$	$C_{13}H_{29}N_2O$	229.2279	3.4	170.15, 141.11, 128.10, 98.06, 84.96, 72.04, 57.07	0.136, -	0.132, -
54	Octanoic acid	$C_8H_{16}O_2$	$C_{13}H_{29}N_2O$	229.2279	4.4	170.15, 141.11, 128.10, 98.06, 84.96, 72.04, 57.07	0.914, -	0.819, -
55	Decanoic acid	$C_{10}H_{20}O_2$	$C_{15}H_{33}N_2O$	257.2593	8.8	198.18, 161.97, 124.87, 109.10, 85.10, 71.08, 60.08	0.242, -	0.781, -
56	Behenic acid	$C_{22}H_{44}O_2$	$C_{27}H_{57}N_2O$	425.4471	24.2	366.37, 321.14, 223.02, 154.12, 135.11, 112.12, 85.10, 60.08	0.326, -	0.8, -

No.	Compound Name	Formula	Derivatization Formula	Precursor ions $[M]^+$	RT (min)	MS/MS	*P* Value and VIP value	
							DM	SM
57	Lignoceric acid	$C_{24}H_{48}O_2$	$C_{29}H_{61}N_2O$	453.4784	25.5	394.40, 272.29, 223.07, 188.04, 152.10, 117.03, 89.06, 60.08	0.536, -	0.088, -

Table S3. Correlation between symptoms improvement and change in metabolites concentration

Variables (Δ)	*r*	*P* Value
	Δ Overall VAS	
5,9,12-octadecatri	-0.2725	0.0197*
ALA	-0.2679	0.0219*
	Δ Runny nose	
15(S)-HETE	0.2394	0.0414*
11(S)-HETE	0.2346	0.0457*
	Δ Blocked nose	
7,10,13-Eicosatrie	-0.2759	0.0181*
	Δ Itchy nose	
9(S)-HPODE	-0.2510	0.0322*
Stearic acid	-0.2315	0.0488*
	Δ Eye symptoms-VAS	
8(S)-HETE	0.3044	0.0088**
5(S)-HETE	0.2878	0.0135*
15(S)-HETE	0.2589	0.0270*
11(S)-HETE	0.2391	0.0417*
	Δ Overall RQLQ	
8(S)-HETE	0.3603	0.0017**
13-HODE or isomer	0.3537	0.0021**
11-dehydro TXB2	0.3743	0.0011**
5(S)-HETE	0.3633	0.0016**
15(S)-HETE	0.4244	0.0002***
11(S)-HETE	0.4181	0.0002***
	Δ Sleep problems	
TXB2	-0.2421	0.0391*
	Δ Non-nose/eye symptoms	
8(S)-HETE	0.2630	0.0246*
13-HODE or isomer	0.2419	0.0392*
11-dehydro TXB2	0.2687	0.0215*
5(S)-HETE	0.2493	0.0334*
15(S)-HETE	0.3183	0.0061**
11(S)-HETE	0.3157	0.0065**
	Δ Practical problem	
8(S)-HETE	0.3892	0.0007***
13-HODE or isomer	0.3081	0.0080***
11-dehydro TXB2	0.3720	0.0012**
5(S)-HETE	0.3741	0.0011**
15(S)-HETE	0.3906	0.0006***
11(S)-HETE	0.3705	0.0013**
	Δ Nose symptoms	
8(S)-HETE	0.3137	0.0069**
13-HODE or isomer	0.3047	0.0088**
11-dehydro TXB2	0.3082	0.0080**
5(S)-HETE	0.3014	0.0096**
15(S)-HETE	0.3286	0.0045**
11(S)-HETE	0.3173	0.0062**
	Δ Eye symptoms-RQLQ	
8(S)-HETE	0.4218	0.0002***
13-HODE or isomer	0.3676	0.0014**
11-dehydro TXB2	0.4101	0.0003***
5(S)-HETE	0.4130	0.0003***
15(S)-HETE	0.4682	0.0000***
11(S)-HETE	0.4481	0.0001***
	Δ Emotional function	

8(S)-HETE	0.2322	0.0480*
13-HODE or isomer	0.3145	0.0067**
11-dehydro TXB2	0.3084	0.0079**
5(S)-HETE	0.2762	0.0180*
15(S)-HETE	0.3547	0.0021**
11(S)-HETE	0.3659	0.0015**

Δ, post-treatment (V2) minus pre-treatment (V0) , *, $P<0.05$; **, $P<0.01$; ***, $P<0.001$
